# Definitive Chemoradiotherapy for Adenocarcinoma of the Esophagus or Esophago-Gastric Junction: A Dutch Population-Based Cohort Study

**DOI:** 10.1245/s10434-026-20428-3

**Published:** 2026-08-24

**Authors:** S. S. G. Gangaram Panday, O. Khoma, H. W. M. van Laarhoven, L. van Doorn, J. Nuyttens, P. S. N. van Rossum, R. H. A. Verhoeven, B. P. L. Wijnhoven, B. Mostert, S. M. Lagarde

**Affiliations:** 1 Department of Surgery, Erasmus MC Cancer Institute, University Medical Center, Rotterdam, the Netherlands; 2 Department of Surgery, Royal Prince Alfred Hospital Institute of Academic Surgery, University of Sydney, Sydney, NSW Australia; 3 Department of Medical Oncology, Amsterdam UMC Location University of Amsterdam, Amsterdam, the Netherlands; 4 Cancer Center Amsterdam, Cancer Treatment and Quality of Life, Amsterdam, the Netherlands; 5 Department of Medical Oncology, Erasmus MC Cancer Institute, University Medical Center, Rotterdam, the Netherlands; 6 Department of Radiation Oncology, Erasmus MC Cancer Institute, University Medical Center, Rotterdam, the Netherlands; 7 Department of Radiation Oncology, Amsterdam UMC Location Free University Amsterdam, Amsterdam, the Netherlands; 8 Netherlands Comprehensive Cancer Organisation (IKNL), Department of Research and Development, Utrecht, the Netherlands

**Keywords:** Definitive chemoradiotherapy, Oesophageal adenocarcinoma, Real-world data, Survival

## Abstract

**Background:**

Locally advanced esophageal adenocarcinoma has a poor prognosis and limited treatment options when surgery is not possible or desired. Although definitive chemoradiotherapy (dCRT) is currently administered as a curatively intended treatment, limited data exist regarding its efficacy for patients with adenocarcinoma of the esophagus, necessitating further evaluation of survival outcomes.

**Methods:**

Patients with a diagnosis of adenocarcinoma of the esophagus or esophago-gastric junction who had undergone dCRT (defined as a radiation dose of > 41.4 Gy and ≤ 50.4 Gy, respectively) were identified from the nationwide Netherlands Cancer Registry (2015–2022). Overall survival (OS) was analyzed, and multivariable Cox regression analysis was performed to evaluate prognostic factors. Progression-free survival (PFS) was estimated for the subgroup of patients with detailed follow-up data from 2015 to 2017.

**Results:**

The study analyzed 926 patients, including 263 patients with data on recurrence patterns. Of these patients, 77% had a diagnosis of stage III/IVa disease, and 72% had a WHO performance status of 0 to 1. The 90-day mortality rate was 7.1%. The median OS was 19.6 months (95% confidence interval [CI], 18.6–21.2 months), and the 3-year OS was 28%. The median PFS was 12.1 months (95% CI, 9.7–14.0 months). Clinical T3–T4 category, cN+ category, WHO performance status, and tumor length were linked to worse survival, although the multivariable model had limited discriminatory power.

**Conclusion:**

Real-world OS and PFS for patients with adenocarcinoma of the esophagus or esophago-gastric junction who received dCRT were poor, and 90-day mortality was substantial. These outcomes highlight the need for optimal patient selection, well-informed shared decision-making, and the development of improved treatment strategies.

**Supplementary Information:**

The online version contains supplementary material available at https://doi.org/10.1245/s10434-026-20428-3.

Esophageal adenocarcinoma (AC) incidence rates are increasing in the Western world.^1^ Approximately half of patients present with locally advanced disease and are candidates for curatively intended multimodality treatment. The highest rates of cure are achieved by the combination of neoadjuvant chemoradiotherapy (nCRT) or perioperative chemotherapy (pCT) and esophagectomy with or without checkpoint inhibitors.^2–6^ Chemoradiotherapy at a higher dose without subsequent esophagectomy, commonly referred to as definitive chemoradiotherapy (dCRT), is an alternative treatment for patients with an unresectable tumor or proximal location of the tumor, patients who are medically unfit for surgery, and those who prefer a nonsurgical approach.^7,8^ As such, it is also used with curative intent for patients who would otherwise have undergone surgery.

During the past decades, dCRT has been optimized by the use of more modern radiation techniques and less toxic chemotherapy regimens.^9,10^ Currently, the most frequently administered dCRT scheme in the Netherlands comprises six cycles of weekly carboplatin (area under the curve [AUC], (2) and paclitaxel (50 mg/m^2^), with radiotherapy to the esophageal tumor and involved nodes using 50.4 Gy in 28 fractions (5 fractions per week).^9–11^

The ARTDECO trial, which investigated the effect of radiation dose escalation, found no benefit in overall survival (OS) with a higher radiation dose to the primary tumor and a 3-year OS of 42% for squamous cell carcinoma (SCC) and AC combined. The 3-year progression-free survival (PFS) was 23% for the subgroup of patients with AC. No data on OS for this subgroup have been published.^12^

A large retrospective study from the United States including patients with AC reported a 5-year OS of 11% for patients unfit for surgery and 14% for those fit for surgery after dCRT. In this population, 83 % received ≥ 50 Gy, and 17% received 41.4–50 Gy.^13^ Other studies also report poor survival after dCRT, particularly for elderly patients (age > 75 years) and those with unresectable tumors, but these studies lack details on the dCRT regimen.^7,14,15^

The main limitations of these studies are variability in dCRT definitions and protocols. An update on the outcomes for patients who have AC treated with contemporary radiation techniques and chemotherapy regimens is warranted to enable optimal treatment decisions and shared decision-making for patients with a potential indication for dCRT.^16^

This study aimed to examine real-world outcomes of dCRT for esophageal adenocarcinoma in a comprehensive nationwide Dutch cohort using a uniform dCRT definition, focusing on OS, 90-day mortality, PFS, and prognostic factors for OS.

## Patients and Methods

### Study Design and Patients

Data were obtained from the nationwide Netherlands Cancer Registry (NCR). The inclusion criteria were based on the standard doses of radiotherapy in the Netherlands, where the standard dosage for dCRT is 50.4 Gy.

The study included patients with a diagnosis of adenocarcinoma (cTanyNanyM0) of the esophagus or esophagogastric junction (OGJ) who received > 41.4 Gy (because 41.4 Gy is the standard nCRT dosage) and ≤ 50.4 Gy combined with up to six weekly cycles of carboplatin and paclitaxel between 2015 and 2022. Initially, the NCR excluded patients with metastatic disease, those who received > 50.4 Gy, and those scheduled for surgery at initial diagnosis (which included most patients treated with neoadjuvant chemoradiotherapy) before the dataset was provided. From the remaining cohort, the study further excluded patients who received a radiotherapy dose ≤ 41.4 Gy as well as those with another malignancy diagnosed within 100 days before the diagnosis. Due to the lack of uniformity in nCRT dosage across hospitals in 2015, patients who received CRT were excluded if they were registered in the NCR as having undergone surgery within the same treatment episode as CRT to further avoid including cases intended as nCRT.

Because the reasons for radiotherapy dose reduction and the intended aim of nCRT versus dCRT were not recorded, to avoid including patients who underwent nCRT instead of dCRT, the study included only those who received > 41.4Gy (i.e., completed or nearly completed radiotherapy up to 50.4 Gy). Survival data were available until 31 January 2025. For patients treated from 2015 to 2017, data on tumor recurrence and salvage treatment also were available.

### Outcomes

The primary outcome was OS, calculated from the end of dCRT to death or last follow-up visit to exclude immortal time bias. Time from diagnosis to the end of dCRT also was reported. The secondary outcomes included OS according to tumor stage, 30- and 90-day mortality after the end of dCRT, PFS (defined as the interval from the end of dCRT to the earliest occurrence of disease progression, either locoregional regrowth or distant dissemination, or until death or last follow-up visit), and details of salvage treatment. Finally, prognostic factors for OS were evaluated. Toxicity data were not available.

### Statistical Analysis

Frequencies and percentages were used to describe baseline clinical and tumor characteristics, as well as details of salvage treatment. Overall survival was analyzed using Kaplan-Meier statistics, with 1- and 3-year survival rates calculated. Survival analyses were performed according to clinical tumor stage, with clinical tumor-node-metastasis (cTNM) staging reported according to the eighth edition of the Union for International Cancer Contro (UICC) staging manual.^17^

Univariable Cox regression was used to calculate hazard ratios (HRs) with 95% confidence intervals (CIs) between tumor stages. Kaplan-Meier statistics were used to analyze PFS survival for the subgroup of patients from 2015 to 2017.

Prognostic factors were evaluated using multivariable Cox regression analysis, studying year of diagnosis, sex, age, WHO performance status, clinical T category, clinical N category, tumor location, and Lauren classification. The assumption of a linear relationship between age and the log hazard was assessed using restricted cubic splines with three degrees of freedom. The proportional hazards assumption was assessed using Schoenfeld residuals. Multiple imputation using the multiple imputation by chained equations (MICE) algorithm was applied to handle missing data in prognostic factors, ensuring that all patients were included in the analysis.^18^ All cTx and cNx classifications were treated as missing values. Results were expressed as hazard ratios with 95% confidence intervals.

Discrimination, assessed using the time-dependent concordance statistic (c-statistic), reflects the model’s ability to distinguish between patients who experience the event (i.e., death) and those who do not. A c-statistic of ≥ 0.7 indicates good discriminatory ability, with values closer to 1 signifying stronger model performance.

To correct for optimism, bootstrapping with 1000 samples was performed, incorporating single imputation within each bootstrap iteration to account for missing data. Additional subgroup analyses were performed to assess the association of radiotherapy dose and number of completed chemotherapy cycles with OS and PFS. Both variables also were incorporated into the multivariable Cox regression models.

Statistical significance was defined as a *p* value lower than 0.05. Data were analyzed using R version 4.2.2 (R Foundation for Statistical Computing, Vienna, Austria).

## Results

**Fig. 1:**
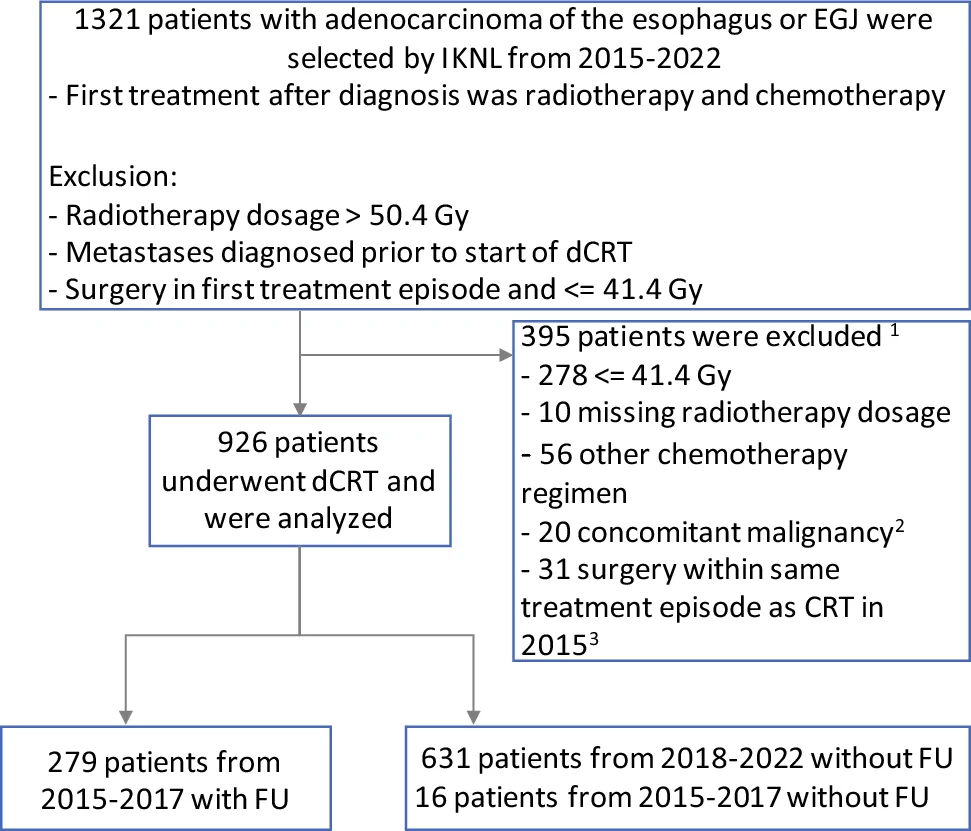
Flowchart of patients. EGJ, esophagogastric junction; NCR, nationwide Netherlands Cancer Registry; dCRT, definitive chemoradiotherapy; FU, follow-up. ^1^Selection was in the order of radiotherapy, chemotherapy, and malignancy. ^2^Diagnosed within 100 days before esophageal carcinoma, considered to have a negative impact on prognosis, and excluded. ^3^In 2015, due to lack of uniformity in nCRT dosage across hospitals, patients with CRT (which could have been intended for nCRT) were excluded if they were registered in the NCR as having undergone surgery within the same treatment episode as CRT

The NCR encompassed 1321 potentially eligible patients during the study period. Of these patients, 278 were excluded due to receiving a radiotherapy dose of ≤ 41.4 Gy and 10 patients due to missing information on radiotherapy dose. Additionally, 56 patients were excluded for receiving a variant chemotherapy regimen, and 20 had a concomitant malignancy. A total of 31 patients were excluded for receiving CRT and surgery in the same treatment episode in 2015. This resulted in a final cohort of 926 patients, of whom 844 (91%) received a dose of 50.4 Gy and 82 received more than 41.4Gy but less than 50.4Gy (Fig. 1).

Altogether, 56% of the patients completed all six cycles of paclitaxel, and 55% completed all six cycles of carboplatin. The 926 patients had a median age of 75 years (interquartile range [IQR], 68–80 years). Stage III/IVa disease was present in 719 patients (77%), and WHO performance status was 0–1 for 662 patients (72%) (Table 1). The median time from diagnosis to start of dCRT was 1.4 months (IQR, 1.1–1.8 months).

**Table 1:** Baseline characteristics of patients

Overall (*n* = 926)			2015–2017 (*n* = 279) *n* (%)
Median age: years (IQR)		75 (68–80)	75.00 (67–79)
Sex	Male	735 (79)	226 (81)
Tumor differentiation grade	1	54 (6)	16 (6)
	2	315 (34)	88 (32)
	3	292 (32)	83 (30)
	Unknown	265 (29)	92 (33)
Lauren classification	Intestinal	455 (49)	129 (46)
	Diffuse	129 (14)	42 (15)
	Mixed	31 (3)	5 (2)
	Indeterminate	41 (4)	15 (5)
	Unknown	270 (29)	88 (32)
Tumor stage^a^	I	7 (1)	3 (1)
	II	173 (19)	55 (20)
	III	540 (58)	154 (55)
	IVa	179 (19)	54 (19)
	Unknown	27 (3.1)	12 (5)
Clinical T category	cT1	11 (1)	5 (2)
	cT2	268 (29)	94 (34)
	cT3	567 (61)	156 (56)
	cT4a	17 (2)	3 (1)
	cT4b	22 (2)	4 (1)
	cTx	41 (4.7)	17 (6)
Clinical N category	cN0	409 (44)	107 (38)
	cN1	340 (37)	117 (42)
	cN2	141 (15)	41 (15)
	cN3	26 (3)	10 (4)
	cNx	10 (1)	4 (1)
Charlson Comorbidity Index	0	273 (30)	69 (25)
	1	273 (30)	101 (36)
	2	179 (19)	53 (19)
	3	88 (10)	28 (10)
	4	37 (4)	7 (3)
	>4	43 (5)	11 (4)
	Unknown	33 (4)	10 (4)
WHO performance status^b^	0	230 (25)	72 (26)
	1	432 (47)	122 (44)
	2	158 (17)	50 (18)
	3	16 (2)	4 (1)
	4	1 (0)	0
	Unknown	89 (10)	31 (11)
Tumor location	Proximal	17 (2)	7 (3)
	Mid	54 (6)	21 (8)
	Distal	714 (77)	220 (79)
	OGJ	118 (13)	19 (7)
	Unknown	23 (3)	12 (4)
Tumor length median: cm (IQR)		5.00 (3.00–6.00)	5.00 (4.00–6.00)
Radiotherapy dosage	50.4	844 (91)	256 (92)
	>41.4 to <50.4	82 (9)	23 (8)
Paclitaxel cycles	<4	41 (4)	9 (3)
	4	98 (11)	28 (10)
	5	271 (29)	85 (31)
	6	515 (56)	154 (56)
	Unknown	1 (0)	0
Carboplatin cycles	<4	41 (4)	9 (3)
	4	98 (11)	28 (10)
	5	275 (30)	88 (32)
	6	511 (55)	154 (55)
	Unknown	1 (0)	0
Year of diagnosis	2015	97 (11)	93 (33)
	2016	84 (9)	79 (28)
	2017	114 (12)	107 (38)
	2018	116 (13)	
	2019	103 (11)	
	2020	120 (13)	
	2021	132 (14)	
	2022	160 (17)	

IQR, interquartile range; WHO, World Health Organization

^a^Clinical tumor-node-metastasis (cTNM) staging is reported according to the 8th edition of the Union for International Cancer Control staging manual

^b^WHO performance score, assessed on a 5-point scale. Higher number indicates greater disability

**Fig. 2:**
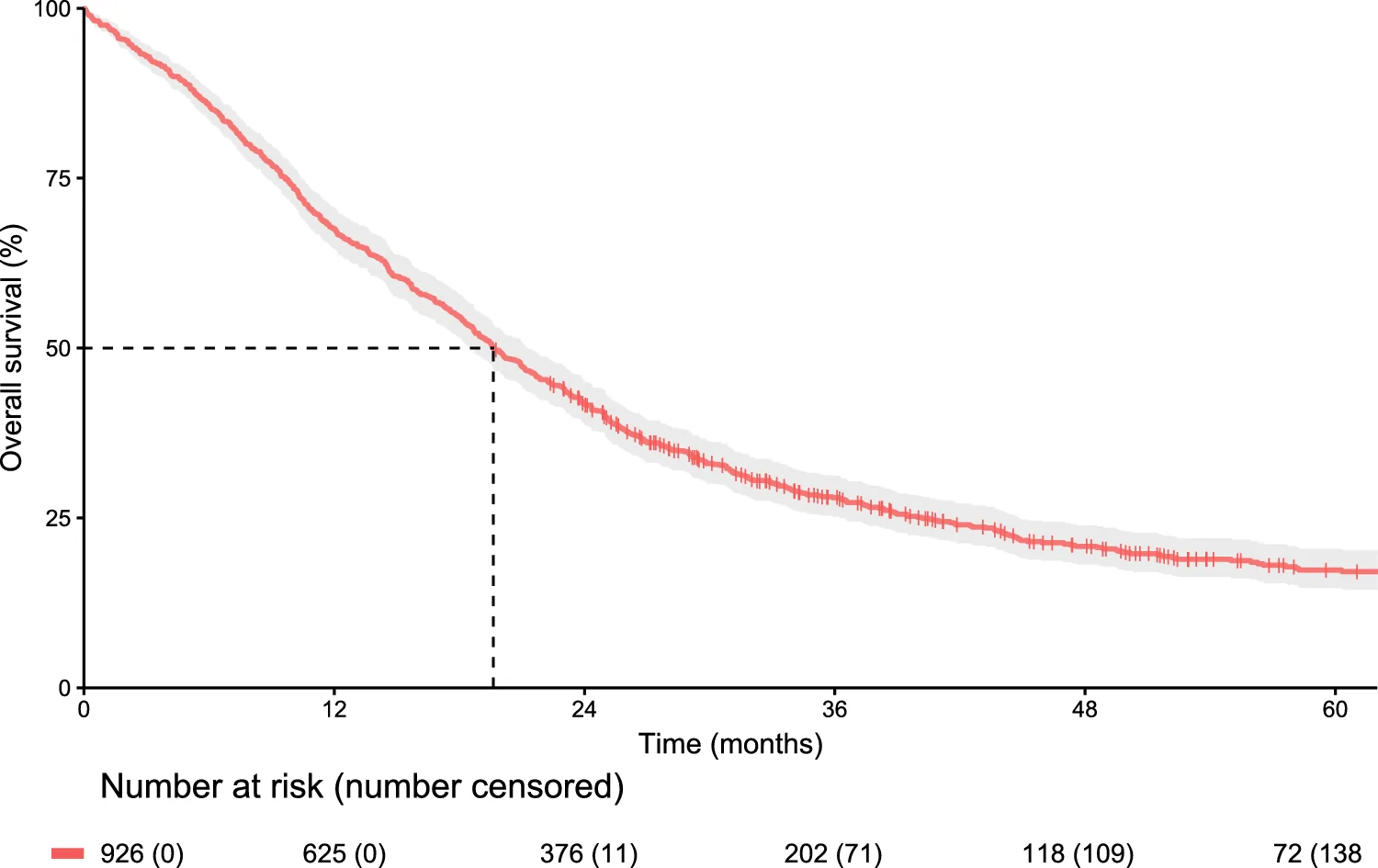
Kaplan-Meier estimates of overall survival. Shaded areas around the curves show 95 % confidence interval

**Fig. 3:**
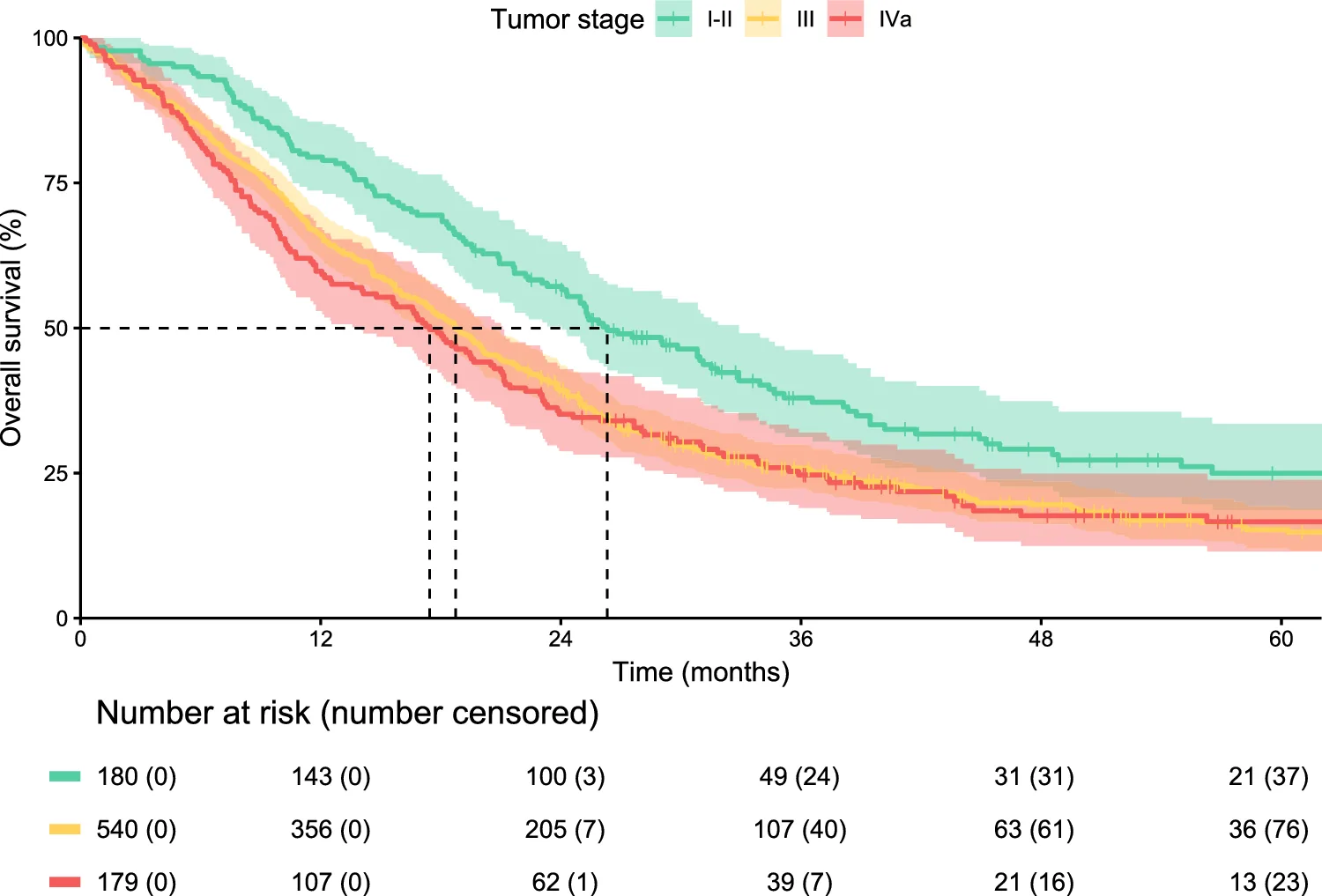
Kaplan-Meier estimates of overall survival according to tumor stage. Shaded areas around the curves show 95 % confidence interval (CI). The study excluded 27 missing. Univariate Cox regression analyses resulted in stage I/II vs stage III (HR, 1.47; 95 % CI, 1.21–1.80) and stage I/II vs stage IVa (HR, 1.50; 95 % CI, 1.18–1.90)

The 30-day post-treatment mortality rate was 2.5% (23/926), whereas the 90-day mortality rate was 7.1% (66/926). After a median follow-up period of 54 months (IQR, 38–79 months), the median OS was 19.6 months (95% CI, 18.6–21.2 months). The 1- and 3-year OS rates were 67.5% (95% CI, 64.5–70.6%) and 28.0% (95% CI, 25.1–31.1), respectively, and the 5-year OS rate was 17.3% (95% CI, 14.7–20.4%) (Fig. 2). In the subgroup analysis of OS by tumor stage, univariable Cox regression analysis showed worse survival for stages III and IVa disease than for stages I and II disease (stage III: HR, 1.47 [95 % CI, 1.21–1.80]; stage IVa: HR 1.50 [95% CI, 1.18–1.90]; Fig. 3).

In the multivariable Cox regression analysis, the factors associated with worse survival included clinical T3–T4 category (cT3–4 vs cT1–2: HR, 1.25; 95% CI, 1.05–1.50; *P* < 0.01), cN+ category (cN+ vs cN0: HR, 1.21; 95% CI, 1.03–1.42; *P* = 0.02), WHO performance status (WHO 1 vs 0: HR, 1.29 [95% CI, 1.08–1.55; *P* < 0.01]; WHO >1 versus 0: HR 1.38 [95 % CI, 1.11–1.72; *P* < 0.01), and tumor length (per increase in cm: HR, 1.05; 95 % CI, 1.00–1.09; *P* = 0.04) (Table 2). Additionally, female sex was associated with improved survival (HR, 0.76; 95 % CI, 0.63–0.92; *P* < 0.01). The initial c-statistic of the multivariable Cox regression model was 0.60, which dropped to 0.58 after correction for optimism, indicating poor discriminative ability.

**Table 2:** Associations between predictors and overall survival

	Multivariable HR	Lower 95 % CI	Upper 95 % CI	*P* Value
Age				
per decade	1.07	0.98	1.17	0.123
Sex				
Male	Ref			
Female	0.76	0.63	0.92	**0.005**
Lauren classification				
Intestinal	Ref			
Diffuse	1.15	0.94	1.41	0.166
Mixed	1.10	0.70	1.74	0.674
cT Category				
cT1–cT2	Ref			
cT3–cT4	1.25	1.05	1.50	**0.012**
cN Category				
cN0	Ref			
cN+	1.21	1.03	1.42	**0.018**
WHO performance status				
0	Ref			
1	1.29	1.08	1.55	**0.007**
>1	1.38	1.11	1.72	**0.004**
Tumor location				
Distal	Ref			
Proximal-mid	1.22	0.92	1.64	0.172
OGJ	1.24	0.99	1.56	0.062
Tumor length				
per centimeter	1.05	1.00	1.09	**0.040**
Year of diagnosis				
2015	Ref			
2016	0.87	0.64	1.19	0.386
2017	1.17	0.87	1.56	0.296
2018	0.93	0.69	1.25	0.642
2019	0.94	0.69	1.26	0.661
2020	0.75	0.55	1.03	0.073
2021	0.69	0.51	0.94	0.018
2022	0.86	0.64	1.15	0.310

HR, hazard ratio; CI, confidence interval; OGJ, esophagogastric junction

**Fig. 4:**
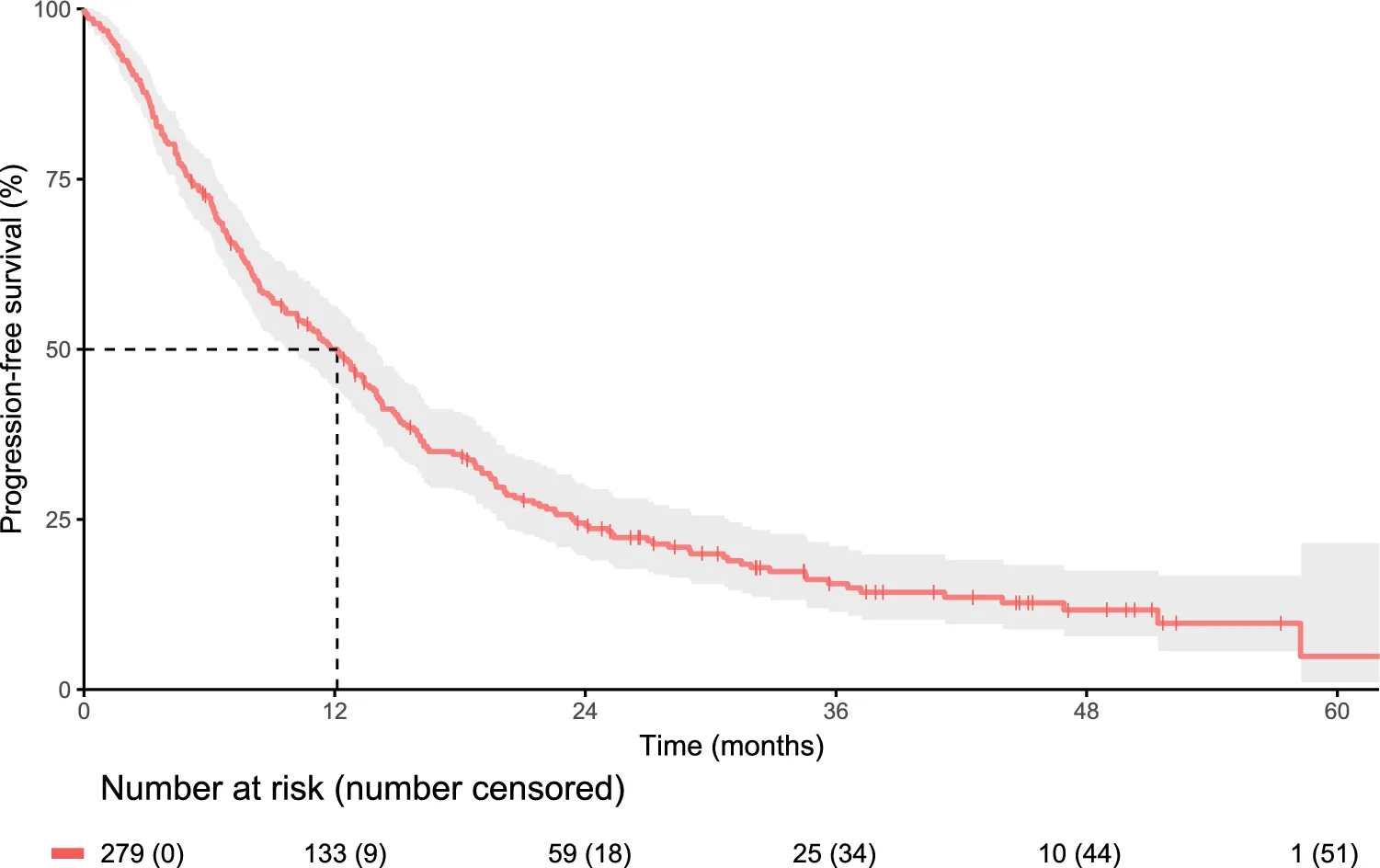
Kaplan-Meier estimates of progression-free survival. Shaded areas around the curves show 95 % confidence interval

Of the 295 patients in the 2015–2017 cohort, for whom data on recurrence date, recurrence location, and salvage treatment were available, 279 (95%) were followed up for disease progression. The remaining 16 patients were not followed up due to follow-up restrictions in specific hospitals. The median PFS was 12.1 months (95% CI, 9.7–14.0 months), with estimated 1-and 3-year PFS rates of 50.0% (95% CI, 44.4–56.3%) and 15.6% (95% CI, 11.5–21.1%), respectively (Fig. 4). At 5 years, only one patient remained at risk.

During the median follow-up period of 41 months (IQR, 27–50 months), 178 (64%) of 279 patients experienced disease progression, 53 (19%) died without documented progression, and 48 (17%) remained event-free. Locoregional progression without distant metastasis occurred for 80 (29%) of 279 patients at a median time of 11.2 months (IQR, 6.9–18.7 months): 63 at the primary tumor site, 2 in isolated regional lymph nodes, and 8 with combined local and regional progression (7 not documented).

The diagnosis for 17 (6%) patients with locoregional progression was determined within 6 months after the end of dCRT. Distant metastases were observed in 97 (35%) of 279 patients at a median time of 8.1 months (IQR, 4.5–15.4 months). Of these patients, 50 had both distant and locoregional progression. The diagnosis for 34 (12%) patients with distant metastases was determined within 6 months after dCRT. One patient had an unknown location of progression (Fig. S1).

Treatment approaches varied based on progression type. Among 97 patients with metastatic disease, 15 underwent stent placement, 21 received radiotherapy, and 20 were treated with systemic therapy, of whom 2 received combined radiotherapy and systemic therapy. No treatment was recorded for the remaining 41 patients.

Among 80 patients with locoregional progression, 12 underwent salvage esophagectomy, all with R0 resection. Stent placement was performed for 29 patients, whereas 16 received radiotherapy, and 6 underwent systemic therapy, of whom 2 were treated with combined radiotherapy and systemic therapy. No treatment was recorded for the remaining 17 patients. Additionally, seven patients underwent esophagectomy after dCRT without evidence of locoregional progression. An unknown site of disease progression and no recorded treatment were noted for one patient.

Additional subgroup and multivariable analyses demonstrated no significant association of radiotherapy dose or completion of chemotherapy cycles with OS or PFS (Figs. S2 to S5).

## Discussion

This nationwide population-based study provides a detailed assessment of outcomes after dCRT for patients with esophageal or OGJ adenocarcinoma using a uniform treatment regimen and contemporary radiotherapy techniques. The 3-year OS was 28%, and the 5-year OS was 17%. These data reflect the real-world effectiveness of dCRT in this patient population and offer an important update to existing literature, particularly because prior studies often included mixed histology or heterogeneous treatment protocols.^13–15^

Our observed 5-year survival rate of 17% is higher than previously reported dCRT outcomes for AC (11–14%), but remains lower than those reported for SCC (22%).^13^ A recent trial comparing high-dose versus standard-dose (50.4 Gy) dCRT in SCC reported a 3-year OS of 38%, underscoring that dCRT remains a good option for SCC.^19^ These differences highlight the need for histology-specific treatment strategies, particularly because adenocarcinoma currently accounts for the majority of esophageal cancers in Western countries.

In comparison with other curative treatment modalities, survival outcomes for dCRT were substantially lower in this cohort. Multimodality therapy with surgery remains the current standard of care for resectable AC. Large randomized controlled trials and real-world data consistently report 3-year OS rates higher than 45 % for patients undergoing nCRT followed by esophagectomy.^2,14,20,21^ In recent Dutch real-world data, 3-year OS was 48% with multimodality therapy including resection.^21^ These figures provide benchmarks for interpreting dCRT outcomes while acknowledging that patients differ in comorbidity burden, performance status, and tumor resectability, reducing or eliminating eligibility for surgery.

For the patients in this cohort, the reason for choosing dCRT was unknown. Therefore, we could not conclude how many patients were unfit for surgery. In a single-center study, nearly one fourth of the patients were treated with dCRT for this reason, although such patterns may not reflect nationwide practice.^7^ When dCRT is chosen to avoid surgical risks, treatment-related toxicity and tumor-related complications must be considered. In this cohort, 90-day mortality was 7.1%, comparable with previous retrospective studies reporting 90-day mortality near 10%, although definitions of the start point to calculate 90-day mortality varied (treatment start vs completion).^7,22^ Although not a direct measure of toxicity, 90-day mortality is a meaningful composite outcome in esophageal cancer, capturing early treatment-related risks.^23^ Given the reported 90-day mortality, patients should be adequately informed about the potential harms of dCRT and available palliative alternatives.

To exclude patients treated with nCRT and planned resection, the study included only those who received > 41.4 Gy of radiotherapy. Possibly up to 10% of the patients who initiated dCRT discontinued treatment early before reaching this threshold of 41.4 Gy, presumably due to toxicity or clinical deterioration, and these were excluded from the current analysis.^7^ Thus, outcomes of all patients starting dCRT might be slightly poorer than described in this cohort. Although data on toxicity were not available, fewer than 60% of the patients completed all six chemotherapy cycles.

A key limitation of this study was the absence of data on the rationale for choosing dCRT over surgery-based approaches. Because the NCR does not document treatment intent or clinical decision-making factors, it remains unclear whether patients received dCRT due to medical inoperability, tumor unresectability, patient preference, or logistical/resource considerations. This limits the ability to interpret outcomes relative to alternative strategies. For instance, patients who decline surgery despite being technically operable may have different prognostic characteristics than those deemed unfit due to comorbidity or tumor stage.

Another limitation was that follow-up data were available only for earlier years, and potential registration errors for salvage esophagectomy in the NCR dataset in 2015 may have led to an underestimation of the number of procedures. Furthermore, we did not have information on Siewert subclassification and were therefore unable to distinguish between OGJ subtypes. Although dCRT in the Netherlands is generally restricted to esophageal cancer and Siewert type I/II tumors, some misclassification cannot be excluded. Other limitations included the retrospective design, selection of the group that had completed or nearly completed dCRT, and lack of data on cause of death and toxicity.

Some of the limitations were balanced by the prospective rigorous data collection, nationwide inclusion, and large sample size. Excluding patients who received ≤ 41.4 Gy of radiation allowed the study to focus on assessing oncologic outcomes specific to AC rather than tolerance of dCRT. Detailed information on recurrence patterns in the subgroup that had follow-up details available may have substituted the cause of death in terms of assessing treatment outcomes. Other strengths of the study were the nationwide standardized protocol of dCRT and the robust collection of survival data and comprehensive information on recurrence patterns and salvage treatment. Future prospective studies or registry enhancements that capture the context of decision-making would allow for more sophisticated stratification and comparative effectiveness analyses.

## Conclusion

Patients with adenocarcinoma of the esophagus or OGJ who have completed or nearly completed dCRT had limited real-world OS and PFS. Male sex, poorer WHO performance status, high tumor category, and node-positive disease were associated with worse outcomes. Based on the results of this study, dCRT should remain reserved for patients with esophageal or OGJ adenocarcinoma who are unable to undergo surgery and should not be offered as a primary curative treatment option.

## Supplementary Information

Below is the link to the electronic supplementary material.

Supplementary file1 (DOCX 86 KB)

## Data Availability

This study was based on the Netherlands Cancer Registry data. The authors do not own these data and hence are not permitted to share them in the original form (only in aggregate form, e.g., publications).
