# Supplementary material for: Definitive Chemoradiotherapy for Adenocarcinoma of the Esophagus or Esophago-Gastric Junction: A Dutch Population-Based Cohort Study
Source: Ann Surg Oncol. 2026 Aug 24;33(12):10375–83. doi: 10.1245/s10434-026-20428-3 (PMC13675376; doi:10.1245/s10434-026-20428-3)
Supplement: Supplementary file 1 [file 10434_2026_20428_MOESM1_ESM.docx]

Supplementary materials

|  | Page |
| --- | --- |
| Figure S1 -- Follow-up of patients from the 2015-2017 cohort | 2 |
| Figure S2 -- Kaplan-Meier estimates of overall survival by radiotherapy dosage | 3 |
| Figure S3 -- Kaplan-Meier estimates of overall survival by chemotherapy cycles | 4 |
| Figure S4 -- Kaplan-Meier estimates of progression-free survival by radiotherapy dosage | 5 |
| Figure S5 -- Kaplan-Meier estimates of progression-free survival by chemotherapy cycles | 6 |

48 event-free (5 underwent oesophagectomy without tumor)

53 died without documented progression

97 distant metastases^1^ (2 underwent oesophagectomy without tumor, prior to metastases)

- 15 stent
- 21 radiotherapy
- 20 systemic therapy (2 combined with radiotherapy)
- 41 no therapy documented

80 locoregional regrowth^2^

- 12 oesophagectomy
- 29 stent
- 16 radiotherapy
- 6 systemic therapy (2 combined with radiotherapy)
- 17 no therapy documented

1 progression with unknown location and treatment

279 patients with follow-up from the 2015-2017 cohort

Figure S1 -- Follow-up of patients from the 2015-2017 cohort. ^1^50 with locoregional progression, 4 with unknown locoregional status; ^2^63 primary tumor site, 2 regional lymph nodes, 8 combined, 7 unknown.

Figure S2 -- Kaplan-Meier estimates of overall survival by radiotherapy dosage. Multivariate cox regression with 50.4Gy-group coded as 0 : HR 1.12, 95%CI 0.84-1.94, *p*=0.46. (also not significant with radiotherapy dosage as a continuous variable)

Figure S3 -- Kaplan-Meier estimates of overall survival by chemotherapy cycles. Multivariate cox regression with 6 cycles-group coded as 0 : HR 1.02, 95%CI 0.88-1.19, *p*=0.80. (also not significant with chemotherapy cycles as a continuous variable)

Figure S4 -- Kaplan-Meier estimates of progression-free survival by radiotherapy dosage. Multivariate cox regression with 50.4Gy-group coded as 0 : HR 0.59, 95%CI 0.27-1.30, *p*=0.19. (also not significant with radiotherapy dosage as a continuous variable)

Figure S5 -- Kaplan-Meier estimates of progression-free survival by chemotherapy cycles. Multivariate cox regression with 6 cycles-group coded as 0 : HR 0.84, 95%CI 0.58-1.21, *p*=0.35. (also not significant with chemotherapy cycles as a continuous variable)
